# Synthesis of Tailored Perfluoro Unsaturated Monomers for Potential Applications in Proton Exchange Membrane Preparation

**DOI:** 10.3390/molecules26185592

**Published:** 2021-09-15

**Authors:** Antonio Monopoli, Michele Casiello, Pietro Cotugno, Antonella Milella, Fabio Palumbo, Francesco Fracassi, Angelo Nacci

**Affiliations:** 1 Chemistry Department, University of Bari Aldo Moro, Via Orabona 4, 70125 Bari, Italy; michele.casiello@uniba.it (M.C.); pietro.cotugno@uniba.it (P.C.); antonella.milella@uniba.it (A.M.); francesco.fracassi@uniba.it (F.F.); angelo.nacci@uniba.it (A.N.); 2 CNR-NANOTEC c/o Department of Chemistry, University of Bari Aldo Moro, Via Orabona 4, 70125 Bari, Italy; Fabio.palumbo@cnr.it; 3 CNR—Istituto di Chimica dei Composti Organometallici (ICCOM), Bari Section, Via Orabona 4, 70126 Bari, Italy

**Keywords:** fuel cells, Nafion^®^, PEM, Heck coupling, vinyl substitution, Wittig-Horner reaction

## Abstract

The aim of the present work is the synthesis and characterization of new perfluorinated monomers bearing, similarly to Nafion^®^, acidic groups for proton transport for potential and future applications in proton exchange membrane (PEM) fuel cells. To this end, we focused our attention on the synthesis of various molecules with (i) sufficient volatility to be used in vacuum polymerization techniques (e.g., PECVD)), (ii) sulfonic, phosphonic, or carboxylic acid functionalities for proton transport capacity of the resulting membrane, (iii) both aliphatic and aromatic perfluorinated tags to diversify the membrane polarity with respect to Nafion**^®^**, and (iv) a double bond to facilitate the polymerization under vacuum giving a preferential way for the chain growth of the polymer. A retrosynthetic approach persuaded us to attempt three main synthetic strategies: (a) organometallic Heck-type cross-coupling, (b) nucleophilic displacement, and (c) Wittig–Horner reaction (carbanion approach). Preliminary results on the plasma deposition of a polymeric film are also presented. The variation of plasma conditions allowed us to point out that the film prepared in the mildest settings (20 W) shows the maximum monomer retention in its structure. In this condition, plasma polymerization likely occurs mainly by rupture of the π bond in the monomer molecule.

## 1. Introduction

Proton exchange membrane fuel cells (PEMFCs), also referred as solid polymer fuel cells, are the simplest type of fuel cells, with application spanning from portable power to automotive [1,2,3]. As solid polymer electrolytes, PEMFCs employ a membrane constituted by a perfluorinated polymer bearing acidic groups, working both as an electronic insulator (to keep separate the oxidative and the reductive half reactions) and as a proton conductor.

Among proton exchange membranes for PEM-type fuel cells, Nafion^®^ is still the most used and studied electrolyte due to its high proton conductivity and moderate swelling in water [4,5]. Alongside its many advantages, the main drawback of Nafion^®^ polymers is due to the membrane hydration, which still represents an operative limit for fuel cells, since the temperature must be maintained below 100 °C [6]. Another factor still limiting the commercial availability of PEMFCs is the cost associated to the active materials employed for their fabrication, i.e., Pt-based catalyst and the Nafion^®^ membrane [6,7,8].

In the last few years, scientists have focused their attention on the development of new catalytic systems with the aim of reducing costs through the enhancement of the performance of the catalyst, by lowering the amount of noble metal (essentially Pt) [9] or by using less expensive metals [8,10,11,12]. Nevertheless, only a few efforts have been made in the direction of finding a valid alternative to Nafion^®^ [5,13,14].

Most alternative proton exchange polymeric membranes are perfluorinated polymers bearing sulfonic or sulfonamidic groups. However, their performances are still not sufficient. Since the nature and chemical characteristics of the membranes are strictly connected to the water transport, it is necessary to increase the number of sulfonic groups per monomeric unit in order to achieve a much higher proton conductivity [15]. Fluorophosphonic acid monomers, which have higher chemical and thermal stabilities than sulfonic acid moieties, are still rarely investigated [16,17,18,19,20,21].

Furthermore, for portable applications, the miniaturization of the FC is crucial (micro-FC), and vacuum-based techniques such as plasma-enhanced chemical vapor deposition (PECVD) are highly desirable to produce both the catalyst layer and the proton exchange membrane in the form of thin films [13,22,23,24,25]. In order to be vacuum-compatible, the volatility of the starting monomer for the deposition of the thin film protonic membrane is mandatory.

In most of the works reported in the literature, these membranes are fabricated from the polymerization of trifluoromethane sulfonic acid, chlorosulfonic acid, or vinyl phosphonic acid with different fluorocarbons or vinylbenzenes [26]. However, most of the commercially available acidic precursors have scarce volatility, due to the hydrogen bond or acid/basic interactions, are highly corrosive, and therefore are not adapted for vacuum deposition [27]. Moreover, the presence of a π bond in the monomer structure can represent a preferential way for the chain growth of the polymer in a plasma environment, avoiding the degradation of other functional groups, such as the acidic one, during the polymerization process [27]. Therefore, tailored precursor synthesis is needed as the commercial ones do not meet all the requirements.

With this in mind, we planned several strategies for synthesizing monomers possessing the following features: (i) a sufficient volatility to be used in vacuum polymerization techniques (e.g., PECVD), (ii) sulfonic, phosphonic, or carboxylic acid functionalities to increase the proton transport capacity of the resulting membrane, (iii) both aliphatic and aromatic perfluorinated tags to diversify the membrane polarity with respect to Nafion**^®^**, and (iv) a double bond to facilitate the polymerization under vacuum, giving a preferential way for the chain growth of the polymer [28].

**Scheme 1:**
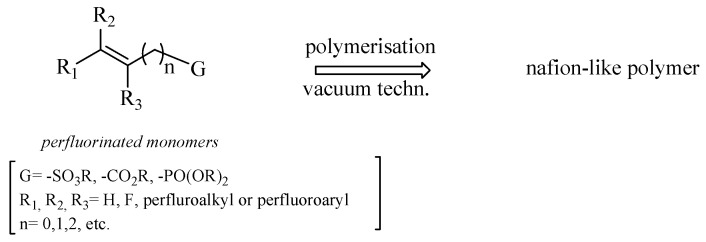
General structure of unsaturated perfluorinated monomers.

On these bases, we focused our attention on perfluorinated esters having the general formula depicted in Scheme 1. After polymerization, the free acidic functionalities responsible for the protonic conductivity can be restored by alkaline hydrolysis of the polymer.

We report here the synthesis and the characterization of new perfluorinated molecules bearing, similarly to Nafion^®^, acidic moieties for the protonic transport, potentially suitable as monomers or co-monomers in PEM fuel cells construction. Finally, we report some preliminary results on the plasma polymerization of one of the synthesized precursors. Since there is a lack of papers dedicated to the synthesis of this type of monomer, our work could represent a valid tool for subsequent applicative research in the preparation of membranes with potential applications in PEM fuel cells.

## 2. Results

### 2.1. Planning the Synthetic Strategy

**Scheme 2:**
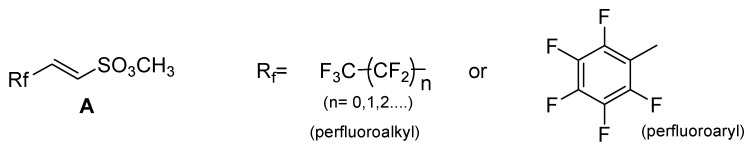
General structure of α,β-unsaturated perfluorinated sulfonic esters.

Initial investigations were directed toward the synthesis of unsaturated perfluorinated sulfonic esters of general formula A (Scheme 2), which to the best of our knowledge has no precedent in the literature [29].

**Scheme 3:**
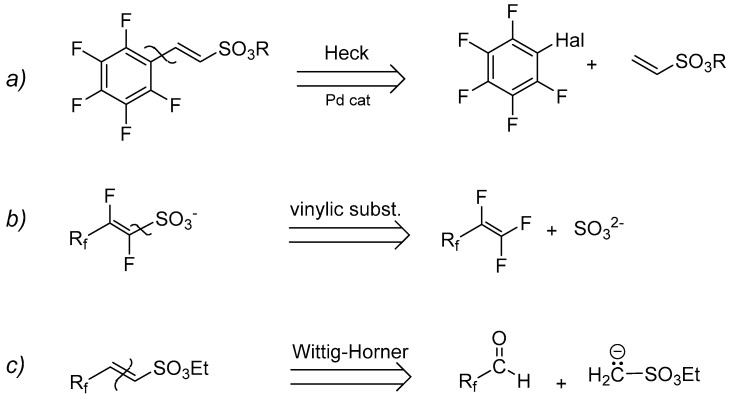
Retrosynthetic routes for the synthesis of perfluorinated sulfonyl monomers (R_f_ = perfluorinated tag).

A retrosynthetic approach persuaded us to attempt three main synthetic strategies (Scheme 3): (a) organometallic Heck-type cross-coupling, (b) nucleophilic displacement, and (c) Wittig–Horner reaction (carbanion approach).

#### 2.1.1. The Heck-Type Coupling Reaction

In the last few decades, we studied several synthetic protocols involving palladium or copper-catalyzed Heck reactions utilizing greener or alternative reaction solvents such as water or ionic liquids [30,31,32]. These latter proved to be particularly effective in the activation of unreactive chloroarenes and hindered olefins [33].

In the present work, since an obvious disconnection analysis results in a direct coupling of a vinyl ester with the iodopentafluorobenzene, the palladium-catalyzed Heck coupling can be considered as an easy and direct way to obtain α,β-unsaturated fluoroaromatic esters. Unfortunately, the lack of papers dealing with the synthesis of these kinds of unsaturated compounds [34] can be considered clear evidence of a difficulty in the Heck coupling when perfluoroaromatic molecules are employed. Moreover, to the best of our knowledge, only two examples were described until now, in which the non-fluorinated analogous phenyl vinylsulfonate (and its sodium salt) have been directly coupled with iodobenzene [35,36].

During the screening, both well-established palladium–phosphine-based complexes and less common but more reactive palladium nanoparticles (PdNPs) were tested as catalysts [30]. In addition, in order to prevent undesirable side reactions such as desulfonation or polymerization processes, we also planned to modify the structure of the target molecule by inserting a spacer between the sulfonate moiety and the double bond. Heck couplings were carried out both in water and in organic solvents, depending on the nature of the starting vinyl substrate.

Obtained results are summarized in Table 1. Unfortunately, reactions performed in organic solvents were unproductive, even in the presence of Pd colloids (Table 1, runs 1–4). GC-MS analyses revealed only the presence of unreacted iodopentafluorobenzene and the disappearance of the vinyl substrate, which may be due to a plausible palladium-catalyzed degradation or a thermal desulfonation of the starting material. 

On the contrary, encouraging results emerged by using vinylsulfonate salt **3** instead of the corresponding ester, under typical phase transfer catalysis conditions. In fact, following a protocol of ours [37], Pd colloids were used in an aqueous solution of tetrabutylammonium hydroxide (TBAOH), affording a 50% of conversion into the coupled product **10**, as revealed by the ^1^HNMR spectrum of the reaction mixture (Table 1, run 5 and Figure S2).

However, all attempts made to raise conversions were unsuccessful, and even the isolation of **10** from the reaction mixture led to very difficult results. Furthermore, no improvements were found even with the less substituted and more reactive 3,4,5-trifluoroiodobenzene (Table 1, run 6). Lower yields were also obtained in the presence of a methylene spacer between the sulfonic group and the double bond (Table 1, run 7).

Then, few modifications were made, and the substitution of fluorine atoms on the aromatic ring, with a perfluorinated tag to the *p*-position, provided satisfactory results only in the case of phenyl sulfonate **14**, which was isolated in a 65% of yield (Table 1, runs 9).

**Scheme 4:**
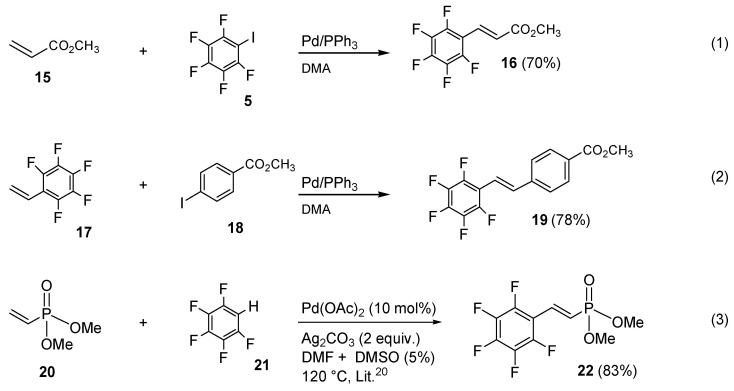
Heck strategy applied to other fluorinated vinyl substrates.

Much better results were obtained with carboxylic esters. Thus, the Heck coupling of methyl acrylate **15** with perfluorinated iodobenzene **5** afforded smoothly (E)-methyl-3-(perfluorophenyl)acrylate **16** in 70% of isolated yield (Scheme 4, Equation (1)). Similar satisfactory results were also reached by coupling pentafluorostyrene (**17**), which afforded methyl 4-(perfluorostyryl)benzoate **19** in 78% of yield (Scheme 4, Equation (2)).

Unfortunately, under similar conditions, all the attempts made to synthesize aryl vinyl phosphonate were unproductive. Therefore, we paid attention to an alternative strategy based on the oxidative Heck coupling by adopting a known procedure of Caryl-H activation for the direct olefination of highly electron-deficient perfluoroarenes catalyzed by Pd(OAc)2 and silver carbonate as a re-oxidant [38]. By means of a slight modification of this method, commercially available vinyl diethyl phosphonate **20** and pentafluorobenzene **21** were coupled for the first time in good yield, affording (E)-dimethyl 2-(perfluorophenyl)vinylphosphonate **22** (Scheme 4, Equation (3)).

In summary, although the organometallic approach can be considered a short and quick way to synthesize olefinic substrates, under explored conditions, it proved to be poorly effective, affording low to moderate yields and showing some drawbacks that could limit the scale up (e.g., toxic solvents and expensive reagents). Studies are in progress to improve these results.

#### 2.1.2. The Nucleophilic Displacement Approach

**Figure 1:**
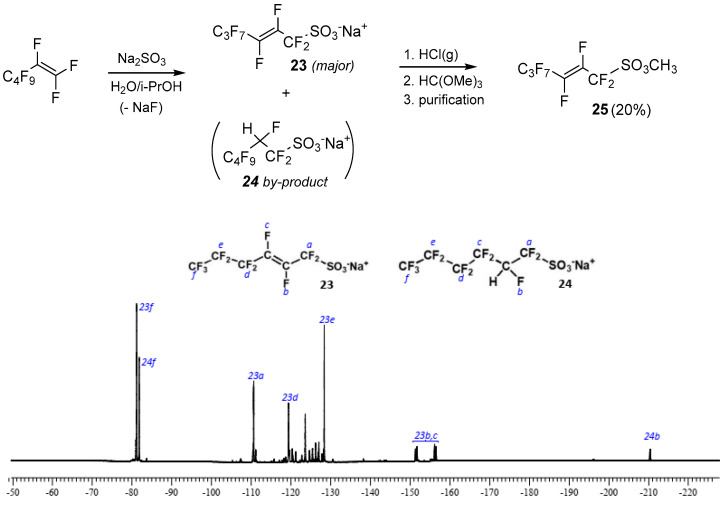
Nucleophilic displacement strategy for sulfonic esters and sulfonate salts **23**–**24** distribution by ^19^F spectrum of the reaction mixture obtained by the treatment of perfluorohex-1-ene with SO_3_^2−^. In the spectrum, the following assignments are possible according to the literature [39]: CF_3_- triplets (23f and 24f) at δ-81.22 ppm, intensity 1 (allylic product **23**) and δ-81.86 ppm, intensity 0.5 (addition product **24**); -CF = CF- at δ−153.83 ppm (23b,c), AB spin system, J(AB) 139.8 Hz; -CFH- at δ−210.44 ppm, ^2^J_F,H_ 30.5 Hz (24a); -CF_2_-S- at δ-110.6 ppm (23a); -CF_2_- at δ−119.40 ppm and 128.44 ppm (23d and 23e, respectively). The CFH group of the addition product creates a magnetic inequivalence of neighbor CF_2_-groups (-CF_2_-CFH-CF_2_-SO_3_Na) and thus AB spin systems (δ from −108 to −127.5 ppm), which has not been specified in detail (signals attributable to fluorine atoms 24a,c,d,e).

A different strategy was adopted involving the direct functionalization of terminal perfluoroalkenes with a sulfonic group through the nucleophilic displacement of a fluorine atom attached to the double bond. Based on results by Gross and Engler [39], such a vinylic substitution can be carried out smoothly with sulfite anion, but it occurs preeminently with the double bond rearrangement, affording the allyl sulfonate as a product with only trace amounts of the expected α,β-unsaturated compound. With this in mind, perfluoro-hex-2-ensulfonate **23** was initially prepared by reacting perfluorohex-1-ene with sodium sulfite in water/isopropanol (Figure 1). Unfortunately, this strategy also suffered from the drawback of giving high rates of addition to the double bond as a side reaction, which leads to minor amounts (25% ca.) of the saturated sulfonic salt **24**, as revealed by ^19^F-NMR spectrum in Figure 1.

To obtain highly volatile compounds, salts **23** and **24** were transformed into the corresponding methyl esters by treatment of the reaction mixture with gaseous HCl followed by the addition of trimethylortoformate [40]. After distillation, sulfonate derivative **25** was only obtained in 20% of overall yield (see Experimental section). It is worth mentioning that the addition of compound **24** was the sole by-product observed with this strategy; no compounds coming from the addition of HCl to the double bond were detected during the successive steps of esterification, which was most probably due to the presence of fluorine atoms on vinylic carbons [41].

**Scheme 5:**
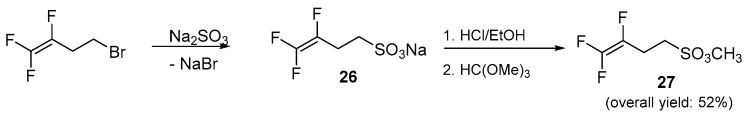
Synthetic strategy for homoallylic fluorosulfonic ester **27**.

To overcome all these problems, the vinylic substitution approach was replaced with a simple nucleophilic displacement on a saturated carbon atom bearing a good leaving group such as a bromide. In addition, for a further increase in reactivity, fluorine atoms near the leaving group were replaced by hydrogen atoms starting from a homoallylic bromide fluorinated onto the double bond, namely 4-bromo-1,1,2-trifluorobut-1-ene, as shown in Scheme 5. More encouraging results were achieved by means of this new strategy and the nucleophilic displacement occurred smoothly enabling the synthesis of homoallylic fluorosulfonate ester **27** in a 52% of overall yield (Scheme 5).

Due to the practical importance of this compound, which seems to attract quite a bit of interest in patents and has no precedent in the literature, the method was validated performing a scale-up experiment on a gram scale, obtaining 20 g of the desired product.

#### 2.1.3. Carbanion Approach: The Wittig–Horner Reaction

**Figure 2:**
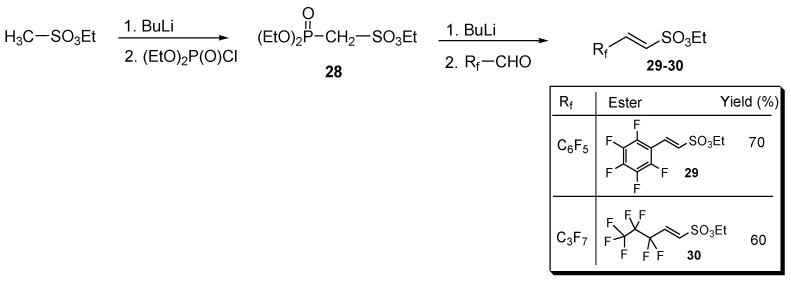
The Wittig–Horner route for synthesizing both aliphatic and aromatic perfluorinated α,β-unsaturated sulfonic esters **29** and **30** (yields in the table are referred to the second step).

The previous strategies enabled the synthesis of perfluorinated aromatic, allylic, and homoallylic sulfonates but failed in the case of their α,β-unsaturated analogous. To overcome this limitation, a literature procedure [41] was adopted based on the Wittig–Horner condensation of ethyl diethylphosphoryl methanesulfonate **28** and a proper perfluorinated aldehyde R_f_-CHO (Figure 2).

In particular, diester (**28**), generated from the commercially available ethyl methansulfonate, was condensed with pentafluorobenzaldehyde and with 2,2,3,3,4,4,4-heptafluorobutanal, affording the perfluoroaryl and perfluoroalkyl sulfonyl esters **29** and **30**, respectively (Figure 2).

In turn, methanesulfonate **28** intermediate was prepared by reacting the carbanion to the alpha position of ethyl methanesulfonate with ethyl chlorophosphite (see for details Section S7 in the Supplementary Materials).

**Figure 3:**
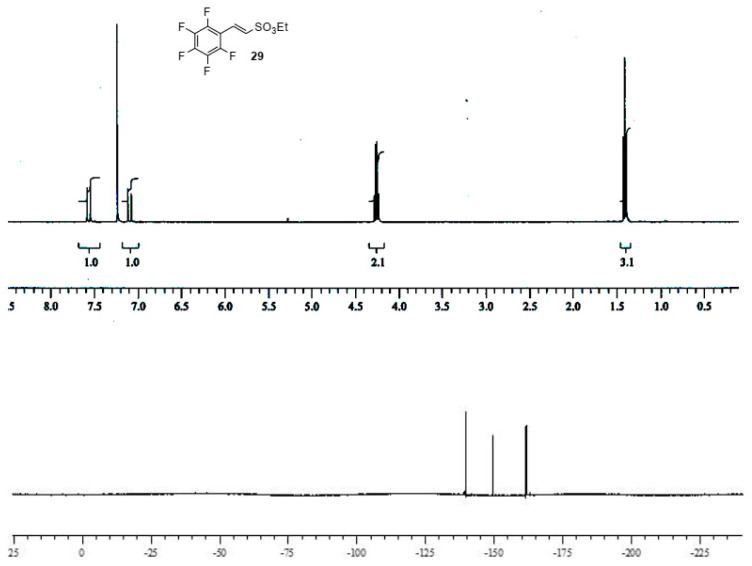
^1^H- and ^19^F-NMR spectra images of (E)-ethyl 2-(perfluorophenyl)ethenesulfonate **29**.

**Figure 4:**
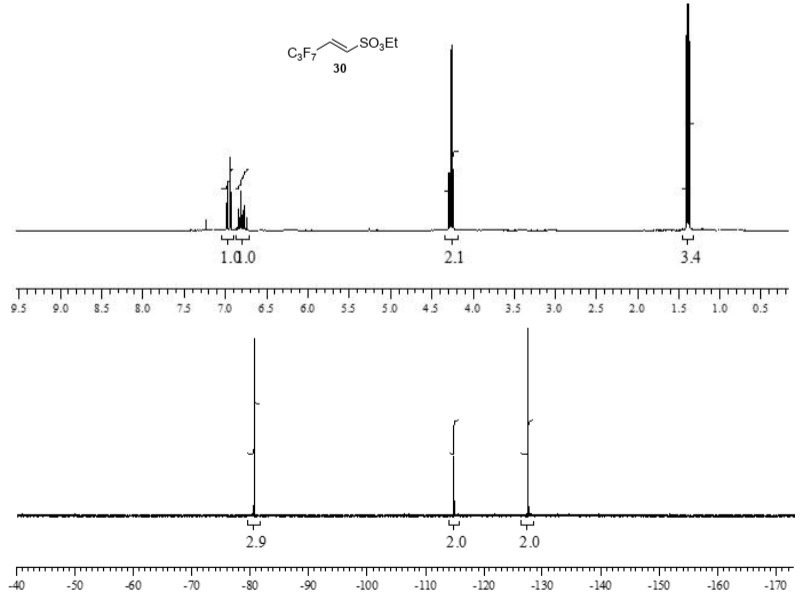
^1^H- and ^19^F-NMR spectra images of (E)-ethyl 3,3,4,4,5,5,5-heptafluoropent-1-ene-1-sulfonate **30**.

Sulfonic esters **29** and **30** were obtained in moderate to good yields as a white solid (m.p. 65 °C) and an oil (b.p. 135 °C, 10 mmHg), respectively, and were completely characterized as reported in the Supplementary Material (see Section S7). Compounds were identified by ^1^H- and ^19^F-NMR spectra (Figure 3 and Figure 4).

**Figure 5:**
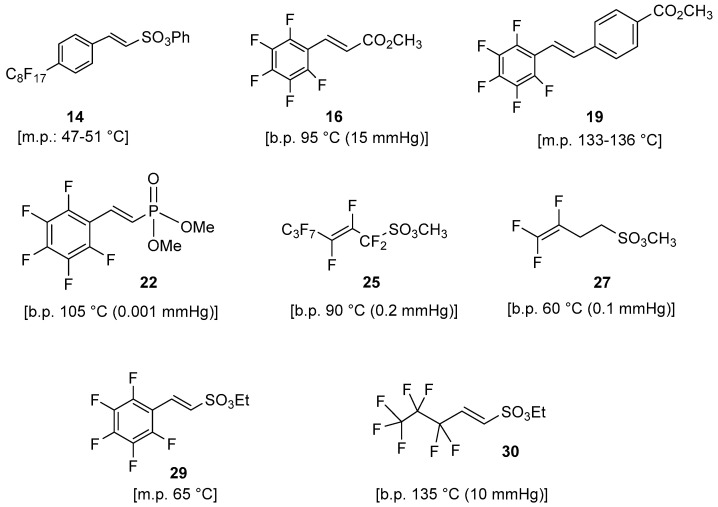
Summary of synthesized perfluorinated monomers.

Below, a summary of perfluorinated monomers synthesized with the three strategies used in this work is listed (Figure 5).

All these compounds were subjected to a selection based on their volatility for choosing the best candidate as suitable monomer for the polymerization using low-pressure deposition techniques.

### 2.2. Thin Film Membrane Deposition by Low-Pressure Plasma

Due to the good thermal stability and high volatility, monomer **27** was chosen as the best candidate for plasma polymerization. Studies are in progress for compounds **25** and **30**.

**Figure 6:**
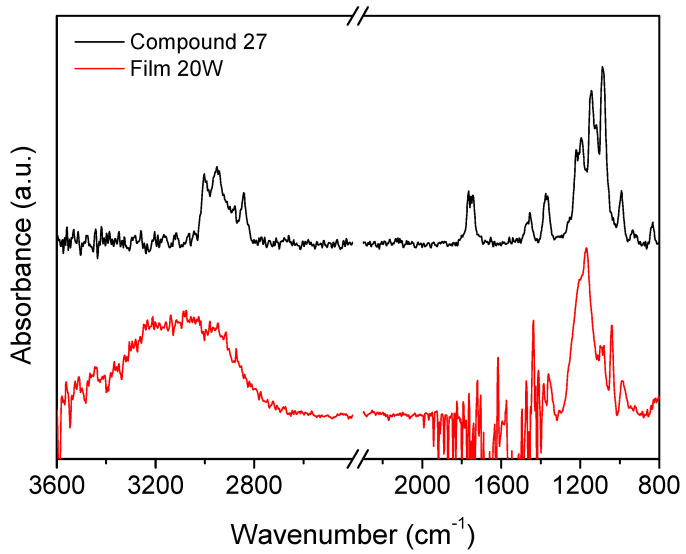
Comparison between infrared spectra of compound 27 vapors and the plasma polymerized film obtained at 20 W.

Figure 6 compares the infrared spectra of vapors of compound **27** and the plasma polymerized film obtained at low power (20 W). The main features of the monomer spectrum are the CH_3_ and CH_2_ absorption bands between 2800 and 3000 cm^−1^, the band at 1767 cm^−1^ assigned to CF_2_=CF, bending of CH_3_ and CH_2_ groups at 1460 cm^−1^. The band at 1370 cm^−1^ is ascribed to S=O stretching in the sulfonic ester group. The broad band 950–1300 cm^−1^ comprises absorptions from CF_2_ groups at about 1200 cm^−1^, the sulfonic ester group at 1143 cm^−1^, and CF at 1090 cm^−1^. Most of these absorptions can be observed also in the spectrum of the film deposited at the lowest plasma power (20 W), namely those of the sulfonic ester functionality and the CF_2_ groups. The retention of such functionalities in the deposited film points to a limited fragmentation of the monomer molecule in the plasma at low power. Instead, the high-wavenumber region is dominated by a broad band from 2800 to 3600 cm^−1^ assigned to OH groups, which was likely formed within the plasma reactor due to some water vapor residues. The CH_3_ and CH_2_ stretching band likely overlaps with the OH one, contributing to further broadening of the latter. Finally, the absorption from the double bond CF_2_=CF in the monomer disappears in the deposited film, indicating that the polymerization reaction has occurred by the rupture of this bond.

**Figure 7:**
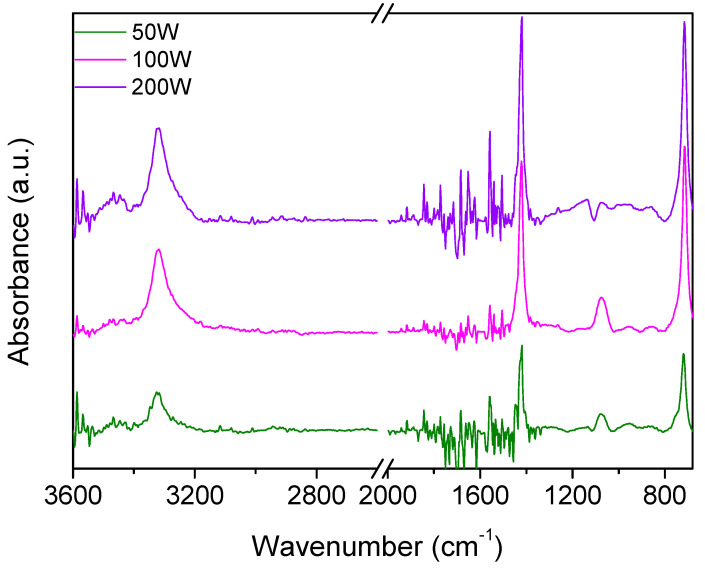
IR spectra of films deposited from compound **27** at variable plasma powers (50–200 W).

Figure 7 shows the infrared spectra of films deposited at increased plasma powers (50–200 W). In this case, major differences can be observed with respect to the spectra of Figure 4. More specifically, the main absorption band between 900 and 1300 cm^−1^ ca. is strongly reduced and less defined, with the only band clearly distinguishable being that at 1080 cm^−1^ assigned to CF groups. The disappearance of absorptions from the π bond, CF_2_, and sulfonic ester groups characterizing the monomer molecule is in agreement with the more energetic plasma environment generated at higher power values. Furthermore, the spectra are characterized by the absorption from OH groups from 3000 to 3500 cm^−1^ and a sharp signal at 1418 cm^−1^, which may be indicative of sulfonyl fluorides or sulfates.

Once more, the formation of these functionalities is reasonable in the energetic plasma environment obtained at higher powers (50–200 W), where new reactive species are formed from the more extensive fragmentation of the precursor molecule. The latter assignment is further confirmed by the increased intensity of the band at higher plasma powers. Finally, the signal at 714 cm^−1^ seems to be correlated to the one at 1418 cm^−1^ and can be assigned to S-F. The increase in the plasma power leads to a more extensive fragmentation of the precursor molecule, and this is reflected in the increase in the hydroxyl group absorption and the increase in the S-F absorptions.

To summarize the information coming from the IR characterization, in accordance with the results reported in the literature, the plasma polymerization of compound 27 led to thin film membranes with high monomer retention only at the lowest plasma powers (20 W). Only in this case, in fact, it is possible to drive the polymerization of the monomer mainly by rupture of the double bond and preserve the sulfonic ester functionality in the deposited film. Increasing the power leads to a more extensive monomer fragmentation, defluorination of the produced radicals, and extensive structural rearrangements in the deposited films.

**Figure 8:**
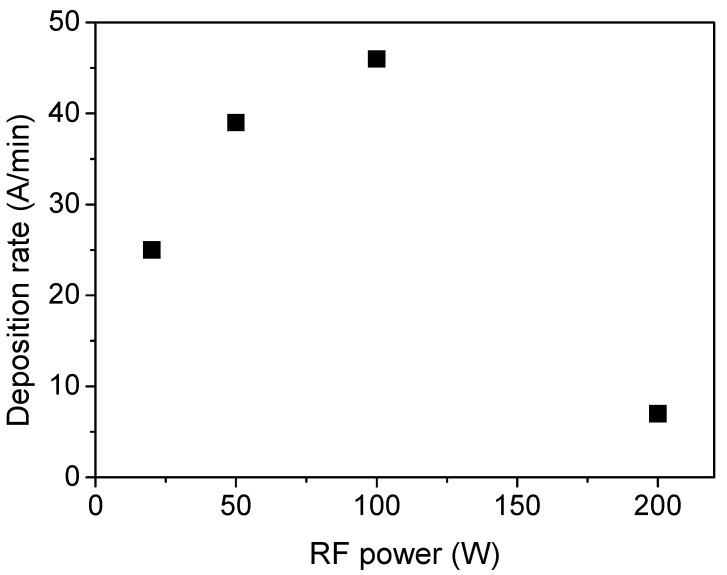
Film deposition rate as a function of the power delivered to the plasma.

Figure 8 reports the film deposition rate, which is determined as the ratio between film thickness and the deposition time (30 min), as a function of the plasma power. The deposition rate increases up to 100 W and then decreases for higher values. This trend is typical in PECVD processes and can be explained considering that below 100 W, increasing the power of the monomer molecule is fragmented to a greater extent in the plasma, thus generating a higher density of radicals, which are precursors of the growing film. This condition is named the activated growth regime. However, above the threshold power, recombination reactions, etching, and sputtering phenomena of the growing film also occur, which results in a reduced film deposition rate (deactivated growth regime) [42].

**Figure 9:**
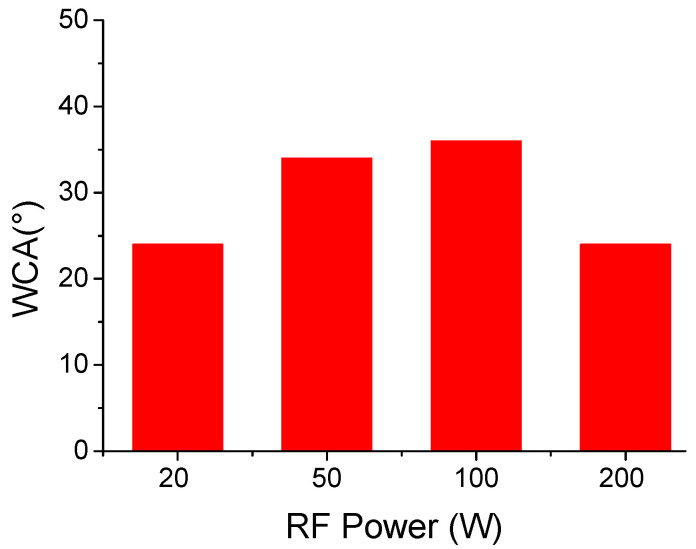
Static water contact angle of deposited films vs. plasma power.

Finally, the wettability of the deposited films was tested through static water contact angle measurements (Figure 9). IR analysis showed that deposited films contain both hydrophilic and hydrophobic moieties, to a different extent, depending on the plasma power used. All deposited films are highly hydrophilic with water contact angles in the range of 25–40°. The film deposited at 20 W with the highest monomer retention is made by hydrophobic moieties such as CF_2_ groups, as well as hydrophilic ones, such as OH functionalities. The hydrophilicity can be likely due to a surface segregation of the polar functionalities. At higher plasma powers, IR spectra point out to an extensive defluorination of the polymer matrix, which can be, beside the presence of the polar functionalities, the reason for the film’s hydrophilicity.

## 3. Materials and Methods

### 3.1. General Remarks

The starting reagents and solvents are commercially available (from Aldrich and ABCR) (see the Supplementary Materials). Solvents (N,N-dimethylformamide (DMA), N,N-dimethylacetamide (DMF), and N-methylpyrrolidone (NMP)) were dried and then distilled before use (see for details Section S1 in the Supplementary Materials).

Pd catalysts of Table 1 were generated in situ from Pd(OAc)_2_ and triphenylphosphine, while Pd(0) nanoparticles catalysts were prepared according to a known procedure by reduction with LiBH_4_ (see: *ChemSusChem* **2012**, *5*, 109–116).

Reactions were monitored by GLC and GC-MS techniques by using an Agilent 5890 A gas-chromatograph and an Agilent 6850/MSD 5975C instruments with a capillary column HP-5MS (Agilent, l. 30 m, i.d. 0.25 mm, s.p.t. 0.25 μ).

NMR spectra were recorded on a Varian Inova 400 MHz spectrometer. Chemical shift values are given in ppm relative to internal Me_4_Si.

Identification of the reaction products was accomplished by their preliminary isolation by column chromatography on silica gel (SiO_2_ 50–200 μm, from Baker) or by distillation. Next, the products were identified by comparison of their MS and NMR spectra with those reported in the literature.

For unknown compounds, high-resolution mass spectra were recorded by using a Shimadzu LCMS-IT-TOF instrument with the following settings: mass range 50–1000 *m/z*, ionization system electrospray ion source in negative ion mode, nebulizer gas nitrogen at 3 bar, dry gas nitrogen at 1.5 L/min and 250 °C, collision gas argon.

### 3.2. Deposition and Characterization of Thin Film Membranes by PECVD

Film depositions were carried out in a cylindrical parallel plate stainless steel reactor evacuated by a turbomolecular/rotary system. Experiments were carried out feeding the plasma with vapors of compound **27** at variable RF power (20–200 W) and a pressure of 500 mTorr. The vapor flow rate was set with a needle valve to 0.25 sccm. The film deposition time was fixed to 30 min, and double-polished silicon was used as a substrate for deposition.

Film chemical composition was investigated by Fourier Transform Infrared (FTIR) spectroscopy (BRUKER, Equinox 55). Spectra were recorded from 400 to 4000 cm^−1^ in absorbance mode at 4 cm^−1^ resolution.

Film water contact angle (WCA) measurements were performed with a manual goniometer (Ramé-Hart, 100) and the reported WCA values were averaged over 5 measurements on each sample with standard deviations of ±1° (for further details see Section S2 of the Supplementary Materials).

### 3.3. General Procedures for the Synthesis of Perfluorinated Monomers

#### 3.3.1. Procedure for the Heck Coupling in Table 1: Synthesis of Perfluorosulfonic Ester 14

In a typical procedure, a 10 mL round-bottomed flask equipped with a magnetic bar was charged with sulfonate ester or salt (**1**–**4**, 0.5 mmol), perfluorohaloarene (**5**–**7**, 0.5 mmol), K_2_CO_3_ (or TBAOH, 1 mmol), Pd acetate (or Pd-colloids, 3 mol%), and PPh_3_ (6 mol%) in 5 mL of DMF (DMA, NMP, or water) and heated at 120 °C for 4 h. At the end of the reaction (GC-MS), the mixture was washed with HCl 5% and extracted with dichloromethane. After drying and evaporation of the solvent in vacuo, the products were purified by silica gel column chromatography (eluent petroleum ether/dichloromethane).

All attempts to isolate the expected products of Table 1 from the reaction mixture (sulfonate **8**,**9**,**12**,**13**,**14**) had unsatisfactory results with the sole exception of phenyl sulfonate **14,** which was obtained in a 65% of yield (see for details Section S3 in the Supplementary Materials).

*(E)-phenyl 2-(4-perfluorooctyl)phenylethene sulfonate* **14**. Pale yellow solid. m.p.: 47–51 °C ^1^H-NMR (400 MHz, CDCl_3_), δ: 6.96 (d, 1 H, *J =* 16.8 Hz, alpha vinyl proton); 7.20–7.42 (m, 5 H, aryl protons SO_3_Ph), 7.55 (d, 1 H, *J =* 16.8 Hz, beta vinyl proton); 7.55 (d, 2 H, *J =* 8.1 Hz, aryl protons); 7.66 (d, 2 H, *J =* 8.1 Hz, aryl protons). HRMS (ESI-TOF) *m/z* [M + H]^+^: calcd for C_22_H_12_F_17_O_3_S^+^ 679.0230, found 679.0238.

#### 3.3.2. Procedure for the Synthesis of Fluorinated Carboxylic Esters 16 and 19 (Scheme 4, Equations (1) and (2))

In a 100 mL round-bottomed flask, equipped with a condenser and a magnetic bar, 1,2,3,4,5-pentafluoro-6-iodobenzene **5** (or methyl 4-iodobenzoate **18**, 5 mmol), methyl acrylate **15** (or 1,2,3,4,5-pentafluoro-6-vinylbenzene **17**, 5 mmol), K_2_CO_3_ (10 mmol), Pd acetate (3 mol%), and PPh_3_ (6 mol%) were refluxed in 50 mL of DMF. At the end of the reaction (GC-MS, 2 ÷ 4 h), the mixture was washed with HCl 5% and extracted with dichloromethane. After drying and evaporation of the solvent in vacuo, the products were purified by silica gel column chromatography (eluent petroleum ether/dichloromethane).

*(E)-methyl 3-(perfluorophenyl)acrylate***16**. Dark red oil, b.p. 95 °C (15 mmHg) (yield 70 %). ^1^H-NMR (400 MHz, CDCl_3_), δ: 3.79 (s, 3H, methoxy); 6.70 (d, 1 H, *J =* 16.5 Hz, alpha vinyl proton); 7.58 (d, 1 H, *J =* 16.5 Hz, beta vinyl proton); ^13^C-NMR (400 MHz, CDCl_3_), δ: 52.2 (MeO), 110.0 (t, ^2^J_C,F_ = 13 Hz), 126.0 (t, ^3^J_C,F_ = 9 Hz), 128.5, 137.95 (d, ^1^J_C,F_ = 253 Hz); 141.81 (d, ^1^J_C,F_ = 258 Hz); 145.75 (d, ^1^J_C,F_ = 254 Hz); 166.52 (carbonyl). ^19^F-NMR (400 MHz, CDCl_3_), δ: −148.05 (dd, 2F, ^3^J_F,F_ = 21 and ^4^J_F,F_ = 6 Hz), −159.78 (t, 1F, ^3^J_F,F_ = 21 Hz), −170.29 (td like, 2F, ^3^J_F,F_ = 21 and ^4^J_F,F_ = 6 Hz); MS (EI): 252 (M^+^, 41), 221 (100), 193 (64), 143 (38), 123 (18), 117 (9), 93 (5), 59 (2). HRMS (ESI-TOF) *m/z* [M + H]^+^: calcd for C_10_H_6_F_5_O_2_^+^ 253.0282, found 253.0271.

*(E)-methyl 4-(perfluorostyryl)benzoate***19**: White solid. ^1^H-NMR (400 MHz, CDCl_3_), δ: 3.90 (s, 3 H, methoxy); 7.02 (d, 1 H, *J =* 16.9 Hz, vinyl proton); 7.40 (d, 1 H, *J =* 16.9 Hz, vinyl proton); 7.53 (d, 2 H, *J =* 8.2 Hz, aryl protons); 8.00 (d, 2 H, *J =* 8.2 Hz, aryl protons); MS (EI): 328 (M^+^, 72), 297 (100), 269 (18), 250 (18), 219 (51), 199 (5), 192 (4), 148 (9), 109 (14), 77 (3), 51 (4). HRMS (ESI-TOF) *m/z* [M + H]^+^: calcd for C_16_H_10_F_5_O_2_^+^ 329.0595, found 329.0590.

#### 3.3.3. Procedure for the Oxidative Heck Coupling: Synthesis of Perfluorinated Phosphonyl Ester 22 (Scheme 4, Equation (3))

This ester was prepared according to the literature [31]. In a 25 mL round-bottomed flask Pd(OAc)_2_ (10 mol%) and Ag_2_CO_3_ (2.0 equiv) under N_2_ were added, which was followed by DMF (2.4 mL) and DMSO (5%, 120 μL) with stirring. Pentafluorobenzene **21** (0.6 mmol, 1.0 equiv) and dimethyl vinylphosphonate **20** (2.0–3.0 equiv) were added subsequently. The mixture was heated at 120 °C (oil bath). After stirring for 12 h, the reaction mixture was cooled to room temperature and diluted with ethyl acetate, washed with 1 N HCl and brine, dried over Na_2_SO_4_, filtered, and concentrated. The residue was purified with silica gel chromatography (petroleum ether:ethyl acetate 3:2 as eluent) to provide the pure product *(E)-dimethyl 2-(perfluorophenyl)vinylphosphonate* **22** as colorless oil (b.p. 105 °C, 0.001 mmHg). ^1^H NMR (400 MHz, CDCl_3_) δ 3.74 (s, 3H), 3.76 (s, 3H), 6.56 (t, *J =* 17.4 Hz, 1H), 7.40 (dd, *J =* 24.2 Hz, 17.4 Hz, 1H). ^13^C NMR δ 52.84, 52.89, 110.38 (m), 123.01 (dt, *J =* 188.5 and 8.4 Hz), 132.78 (d, *J =* 6.1 Hz), 137.95 (dm, ^1^J_C,F_ = 254.8), 141.95 (dm, ^1^J_C,F_ = 257.0 Hz), 145.77 (dm, ^1^J_C,F_ = 255.6 Hz). ^19^F NMR δ -140.0 (dd, ^3^J_F,F_ = 22.0 Hz, ^4^J_F,F_ = 7.3 Hz, 2F), −150.3 (t, ^3^J_F,F_ = 19.5 Hz, 1F), −160.88 (td, ^3^J_F,F_ = 19.5 and ^4^J_F,F_ = 7.3 Hz, 2F). MS (EI) 302 (M^+^, 24), 283 (28), 207 (100), 170 (79), 143 (39), 110 (78), 93 (61). HRMS (ESI-TOF) *m/z* [M + H]^+^: calcd for C_10_H_9_F_5_O_3_P^+^ 303.0204, found 303.0211.

#### 3.3.4. Procedures for the Nucleophilic Substitution: Synthesis of Perfluorinated Sulfonic Esters 25 and 27

Esters **25** and **27** were prepared starting from perfluorohex-1-ene or 4-bromo-1,1,2-trifluorobut-1-ene, respectively, by treatment with sodium sulfite followed by the esterification of sulfonate salts intermediate (**23** and **26**) with trimethyl orthoformate (see for details Scheme 5, Figure 1 and Section S4 in the Supplementary Materials). Sulfonic esters **25** and **27** were characterized as reported below:

*Methyl perfluorohex-2-ene-1-sulfonate***25** was isolated as a red oil (b.p. 90 °C, 0.2 mmHg). ^19^F-NMR (CDCl3) δ ppm (relative to CFCl_3_): −81.20 (t, 3F, ^3^J_F,F_ = 8.20 Hz, CF_3_); −107.22 (dd, 2F, ^3^J_F,F_ = 24.4 and ^4^J_F,F_ = 12.2 Hz, CF_2_-SO_3_), −119.60 (m, 2F, CF_2_-CF_2_-CF=), −128.50 (m, 2F, CF_2_-CF_2_-CF=), −152.50 (dt, 1F, ^3^J_F,F_ = 143.4 and ^3^J_F,F_ = 24.4 Hz, =CF-CF_2_-SO_3_), −153.70 (m, 1F, CF=CF-CF_2_-SO_3_). HRMS (ESI-TOF) *m/z* [M + H]^+^: calcd for C_7_H_4_F_11_O_3_S^+^ 376.9705, found 376.9712.

*Methyl 3,4,4-trifluorobut-3-ene-1-sulfonate***27** as a dark red oil in 89% of yield. ^1^H-NMR (CDCl_3_) δ ppm: 2.67–2.85 (m, 2 H, =CHF-CH_2_), 3.24 (t, 2 H, *J*= 7.3 Hz, -CH_2_-SO_3_CH_3_), 3.83 (s, 3 H, SO_3_CH_3_). ^13^C-NMR (CDCl_3_) δ ppm: 21.05 (d, ^2^J_C,F_ = 22.9 Hz, =CHF-CH_2_), 41.13 (s, -CH_2_-SO_3_CH_3_), 55.96 (s, SO_3_CH_3_). 125.53 (ddd, ^1^J_C,F_ = 235.0 Hz, ^2^J_C,F_ = 53.4 and 18.3 Hz, F_2_C = CHF-), 153.21 (ddd, ^1^J_C,F_ = 288.4 and 276.2 Hz, ^2^J_C,F_ = 45.0 Hz, F_2_C = CHF-). ^19^F-NMR (CDCl_3_) δ ppm: −190.45 (ddt, 1 F, ^3^J_F,F_ = 116.0 and 33.6 Hz, ^3^J_F,H_ = 21.4 = CHF-CH_2_), −135.73 (dd, 1 F, ^2^J_F,F_ = 116.0, ^3^J_F,H_ = 82.4, CF_2_ = CHF-), −116.65 (dd, 1 F, ^2^J_F,F_ = 82.4, ^3^J_F,F_ = 33.6, CF_2_ = CHF-). MS (EI): 204 (0.1), 108 (100), 89 (20), 59 (23), 39 (40). HRMS (ESI-TOF) *m/z* [M + H]^+^: calcd for C_5_H_8_F_3_O_3_S^+^ 205.0141, found 205.0149.

#### 3.3.5. Procedures for Wittig–Horner Reaction: Synthesis of Perfluorinated Sulfonic Esters 29 and 30

These esters were prepared by adapting a literature procedure [35] that exploits the Wittig–Horner reaction (see for details Section S7 of the Supplementary Materials).

*(E)-ethyl 2-(perfluorophenyl)ethenesulfonate***29**. A solution of ethyl diethylphosphoryl methanesulfonate **28** (1.0 equiv) in dry THF (1 mL × 0.25 mmol of **28**) was treated at −78 °C under nitrogen atmosphere with 2.3 M n-BuLi in hexane (approximately 1.05 equiv.). Stirring was continued for 20 min. Then, freshly pentafuorobenzaldehyde (1.1 equiv.) was added. After an additional 45 min. at −78 °C, the solution was allowed to warm to room temperature and stirring was continued for 60 h. The bulk of the solvents was evaporated, and the residue was treated with water (5 mL × 0.25 mmol) and extracted with dichloromethane (3 × 5 mL × 0.25 mmol). The organic layers were dried over MgSO_4_ and evaporated to afford crude unsaturated sulfonyl ester, which was purified by column chromatography (petroleum ether:ethyl acetate 10:1 as eluent) affording pure (E)-ethyl 2-(perfluorophenyl)ethene sulfonate **29** as white solid (m.p. 65 °C) in a 70% of yield. ^1^H NMR (CDCl_3_): 1.40 (t, *J =* 6.9 Hz, 3 H, CH_3_); 4.25 (q, *J =* 6.9, 2 H, CH_2_); 7.09 (d, *J =* 15.9, 1 H, vinyl CH); 7.56 (d, *J =* 15.9, 1 H, vinyl CH). ^19^F NMR (CDCl_3_): −139.7 (dt, ^3^J_F,F_ = 24.4 and ^4^J_F,F_ = 6.1 Hz, 2 F), −149.6 (t, ^3^J_F,F_ = 21.3 Hz, 1 F), −161.8 (m, 2 F). HRMS (ESI-TOF) *m/z* [M + H]^+^: calcd for C_10_H_8_F_5_O_3_S^+^ 303.0109, found 303.0116.

*(E)-ethyl 3,3,4,4,5,5,5-heptafluoropent-1-ene-1-sulfonate***30**. This perfluorinated sulfonic ester was prepared by an analogous procedure from **28** and 2,2,3,3,4,4,4-heptafluorobutanal (CH_2_Cl_2_:petroleum ether 7:3 as eluent) in a 60% of yield. The ester was initially obtained as a mixture of E/Z isomer 85/15 in ratio. After distillation (b.p. 135 °C, 10 mmHg), the major E isomer was isolated as red oil. ^1^H NMR (CDCl_3_): 1.39 (t, *J =* 7.1 Hz, CH_3_), 4.26 (q, *J =* 7.1, 2 H, CH_2_), 6.78 (dt, *J =* 15.4 and 11.2 Hz, vinyl CH), 6.96 (dt, *J =* 15.4 1.5 Hz, 1 H, vinyl CH). ^13^C δ 14.95, 69.01, 108.40 (tm, ^1^J_C,F_ = 264.7 Hz, CF_3_CF_2_-), 112.80 (tt, ^1^J_C,F_ = 255.6 and ^2^J_C,F_ = 32.0 Hz, CF_2_C=), 117.52 (qt, ^1^J_C,F_ = 287.6 and ^2^J_C,F_ = 33.5 Hz, CF_3_), 130.4 (t, ^2^J_C,F_ = 25.2 Hz), 135.8 (t, ^3^J_C,F_ = 8.4 Hz). ^19^F NMR (CDCl_3_): -80.7 (t, ^3^J_F,F_ = 9.2 Hz, 3 F); -114.9 (quintet, ^3^J_F,F_ = 9.2 Hz, 2 F); −127.6 (m, 2 F); HRMS (ESI-TOF) *m/z* [M + H]^+^: calcd for C_7_H_8_F_7_O_3_S^+^ 305.0077, found 305.0071.

## 4. Conclusions

In conclusion, three main synthetic strategies were explored for synthesizing perfluorinated unsaturated sulfonic, phosphonic, and carboxylic compounds to be used as monomers for future applications in producing proton exchange membranes. Among the synthetic pathways presented, the organometallic approach gave the worst results, with low yields, hard isolation of products, and difficult scale up. In contrast, nucleophilic substitution with sulfite anion, as well as Horner–Wittig condensation strategies, gave moderate to good yields depending on the structure of the target molecules. Studies are still underway to improve these results.

Compound **27** was selected as the best candidate for thin film membrane preparation by PECVD, based on its volatility. Plasma polymerization experiments were carried out at different plasma powers (20–200 W). Comparison of the film chemical composition by IR spectroscopy allowed us to point out that the film prepared in the mildest plasma conditions (20 W) shows the maximum monomer retention in its structure. In this condition, plasma polymerization likely occurs mainly by rupture of the π bond in the monomer molecule. Increasing the power leads to a more extensive monomer fragmentation, defluorination of the radicals and extensive structural rearrangements in the deposited films. Future studies will address the deposition of the thin film by the copolymerization of compound **27** with a different long-chain fluorocarbon and the measurement of the proton exchange capabilities of our thin film membranes and their optimization.

## Figures and Tables

**Table 1:**
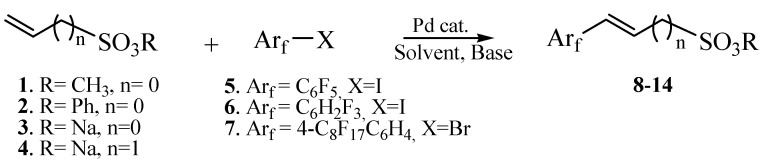
Heck-type coupling of unsaturated sulfonates with perfluoroaryl halides ^a^.

Run	Vinyl Sulfonate	Ar_f_-X	Catalyst	Solvent	Base	Product	Yield (%)
1	**1**	**5**	Pd(OAc)_2_/PPh_3_	NMP	K_2_CO_3_	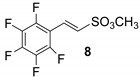	traces
2	**1**	**5**	Pd_colloids_	DMF	K_2_CO_3_		traces
3	**2**	**5**	Pd(OAc)_2_/PPh_3_	DMF	K_2_CO_3_	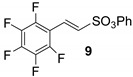	5
4	**2**	**5**	Pd_colloids_	DMF	K_2_CO_3_		traces
5	**3**	**5**	Pd_colloids_	H_2_O	TBAOH	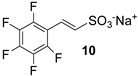	17–50 ^b^
6	**3**	**6**	Pd_colloids_	H_2_O	TBAOH	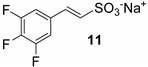	13–45 ^b^
7	**4**	**5**	Pd_colloids_	H_2_O	TBAOH	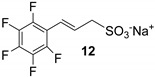	traces
8	**1**	**7**	Pd(OAc)_2_/PPh_3_	DMF	K_2_CO_3_	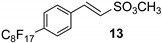	5
9	**2**	**7**	Pd(OAc)_2_/PPh_3_	DMF	K_2_CO_3_	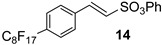	65 ^c^

^a^ Reaction conditions: sulfonate (0.5 mmol), perfluorohaloarene (0.5 mmol), K_2_CO_3_ (or TBAOH, 1 mmol), Pd acetate (or Pdcolloids, 3 mol%), and PPh_3_ (6 mol%) in 5 mL of DMF or water were heated at 120 °C for 8 h (see Supporting Information). ^b^ Unreproducible reactions. Range of yields based on five attempts. Yields were evaluated by ^1^H-NMR of the reaction mixture (see S.I.). ^c^ Isolated yield.

## Data Availability

Not applicable.

## Supplementary Materials

The following are available online.
