# Supplementary material for: Synthesis of Tailored Perfluoro Unsaturated Monomers for Potential Applications in Proton Exchange Membrane Preparation
Source: Molecules. 2021 Sep 15;26(18):5592. doi: 10.3390/molecules26185592 (PMC8470954; doi:10.3390/molecules26185592)
Supplement: Supplementary file 1 [file molecules-26-05592-s001.zip › molecules-1340622-supplementary.pdf]

Supplementary Material

Synthesis of Tailored Perfluoro Unsaturated Monomers for Potential Applications in Proton Exchange Membranes

Antonio Monopoli*, Michele Casiello, Pietro Cotugno, Antonella Milella, Francesco Fracassi, and Angelo Nacci

Contents:

S.1 General Remarks 2

S.2 Thin film membrane deposition by PECVD technique and characterization 2

S.3 Procedure of the Heck coupling for the synthesis of perfluorosulfonyl ester **8-14** *of Table 1* 3

S.4 Procedure for Heck-type preparation of carboxylic esters **16** *and* **19** *(eq. 1-2)*.......................................5

S.5 Procedure for oxidative Heck coupling: synthesis of phosphonyl ester **22** *(scheme 4, eq. 3).* 5

S.6 Procedures for the nucleophilic substitution: synthesis of perfluorinated sulfonic esters **25** *and* **27**......5

S.7 Procedures for Wittig-Horner reaction: synthesis of perfluorinated sulfonic esters **29** *and* **30**.............6

S.1 General Remarks

Starting reagents 2-chloroethanesulfonyl chloride, allyl bromide, phenyl ethenesulfonate **2**, sodium ethenesulfonate **4** (water soln. 25%), 1,2,3,4,5-pentafluoro-6-iodobenzene **5**, tetrabutylammonium hydroxide ($Bu_4NOH \times H_2O$, 40% in H_2O), 1,2,3-trifluoro-5-iodobenzene **6**, 1-bromo-4-perfluorooctylbenzene **7**, methyl acrylate **15**, 1,2,3,4,5-pentafluoro-6-vinylbenzene **17**, methyl 4-iodobenzoate **18**, dimethyl vinylphosphonate **20**, 1,2,3,4,5-pentafluorobenzene **21**, perfluorohex-1-ene, sodium sulfite, trimethyl orthoformate, 4-bromo-1,1,2-trifluorobut-1-ene, ethyl methansulfonate, butyl lithium, ethyl chlorophosphate, pentafluorobenzaldehyde, and 2,2,3,3,4,4,4-heptafluorobutanal are commercially available (from Aldrich and ABCR) and were used as received. Solvents (N,N-dimethylformamide DMA, N,N-dimethylacetamide DMF and N-methylpyrrolidone NMP) were dried and then distilled before to use.

Pd catalysts of table 1 were generated *in situ* from $Pd(OAc)_2$ and triphenylphosphine, while Pd(0) nanoparticles catalysts were prepared according to a known procedure[1] by reduction $Pd(OAc)_2$ with $LiBH_4$.

Reactions were monitored by GLC and GC-MS techniques by using an Agilent 5890 A gas-chromatograph and an Agilent 6850/MSD 5975C instrument, respectively. Both the instruments were equipped with a capillary column HP-5MS (Agilent, l. 30 m, i.d. 0.25 mm, s.p.t. 0.25 μ). NMR spectra were recorded on a Varian Inova 400 MHz spectrometer. Chemical shift values are given in ppm relative to internal Me_4Si.

Identification of the reaction products was accomplished by their preliminary isolation by column chromatography on silica gel (SiO_2 50-200 μm, from Baker) and by distillation. Next, the products were identified by comparison of their MS and NMR spectra with those reported in the literature.

For unknown compounds, high-resolution mass spectra were recorded by using Shimadzu LCMSIT-TOF instrument with the following settings: mass range 50-1000 m/z, ionization system electrospray ion source in negative ion mode, nebulizer gas nitrogen at 3 bar, dry gas nitrogen at 1.5 L/min and 250 °C, collision gas argon.

S.2 Deposition and characterization of thin film membranes by PECVD

Film depositions were carried out in the cylindrical parallel plate stainless steel reactor shown in Figure **S1**, evacuated by a turbomolecular/rotary system.

The upper shower electrode (15 cm in diameter), was connected to a radiofrequency (RF, 13.56 MHz) power supply (Cesar 1310, Dressler), through an impedance automatic matching unit (ULVAC, model 002A). The lower grounded electrode (19 cm in diameter) was set 5 cm apart from the upper RF electrode and acted as sample holder. The pressure in the chamber was monitored by a capacitive Baratron (MKS Instruments) and it was fixed at 500 mTorr. Experiments were carried out feeding the plasma with vapours of compound 27 at variable RF power (20 - 200 W). The vapour flow rate was set with a needle valve to 0.25 sccm.

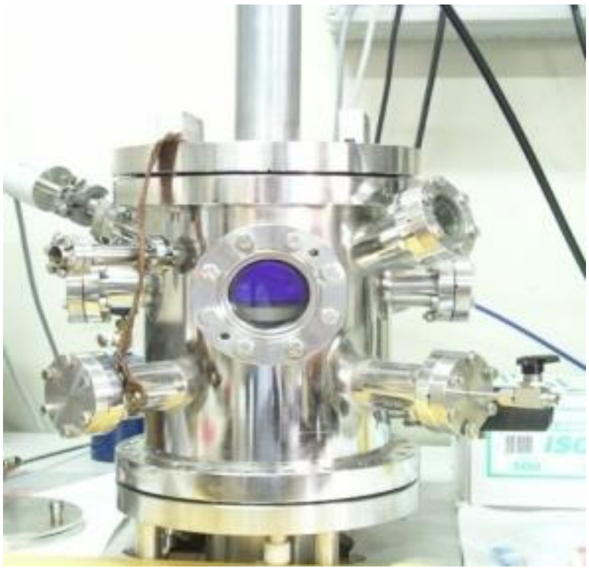

Figure S1. Low pressure plasma reactor used for plasma polymerization of compound 27.

Film deposition time was fixed to 30 min and double-polished silicon was used as substrate for deposition.

Film chemical composition was investigated by Fourier Transform Infrared (FTIR) spectroscopy (BRUKER, Equinox 55). Spectra were recorded from 400 to 4000 cm^{-1} in absorbance mode at 4 cm^{-1} resolution. In order to minimize effects of water vapor and carbon dioxide, the spectrometer was purged with nitrogen for 15 min between each measurement. After baseline correction spectra were normalized to film thickness measured by a stylus profiler (KLA-Tencor, AlphaStep D-120).

Before film deposition, the FTIR spectrum of the vapour of compound 27 was acquired by putting the instrument online with the plasma reactor. More specifically, the parallel beam exiting the spectrometer

[1] A. Banerjee, R. Theron, R. W. J. Scott *ChemSusChem* **2012**, *5*, 109-116.

from a KBr window, is directed through a ZnSe window (transmission range: from 20000 to about 700 cm^{-1}) into the reactor feed with vapours. Finally the beam is focused with a gold coated paraboloid mirror onto a Mercury Cadmium Telluride (MCT) detector (spectral range: 5000-500 cm^{-1}) cooled with liquid N_2. The optical path is purged with N_2 in order to remove CO_2 and water vapour. Infrared spectrum of gas phase was recorded from 500 to 4000 cm^{-1} with a spectral resolution of 1 cm^{-1} over 250 scans.

Film water contact angle (WCA) measurements were performed with a manual goniometer (Ramé-Hart, 100) and the reported WCA values were averaged over 5 measurements on each sample.

S.3 Procedure of the Heck coupling for the synthesis of perfluorosulfonyl ester 8-14 of Table 1

The synthesis of sulfonic esters **8-14** listed in Table 1 was attempted by a Heck-type procedure between a terminal unsaturated sulfonates ester (or its sodium salt) **1-4** and perfluoroaromatic halides **5-7**, as depicted below:

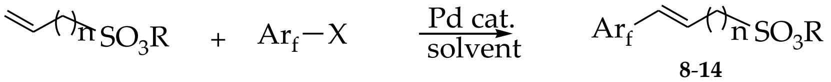

8-14

1. R= CH_3, n= 0
2. R= Ph, n= 0
3. R= Na, n=0
4. R= Na, n=1

5. $Ar_f = C_6F_5$, X=I
6. Ar_f = 3,4,5-$F_3C_6H_2$, X=I
7. Ar_f = 4-$C_8F_{17}C_6H_4$, X=Br

8. R= CH_3, $Ar_f = C_6F_5$, n= 0
9. R= Ph, $Ar_f = C_6F_5$, n= 0
10. R= Na, $Ar_f = C_6F_5$, n=0
11. R= Na, 3,4,5-$F_3C_6H_2$, n=0
12. R= Na, $Ar_f = C_6F_5$, n=1
13. R= CH_3, Ar_f = 4-$C_8F_{17}C_6H_4$, n=0
14. R= Ph, Ar_f = 4-$C_8F_{17}C_6H_4$, n=0

General procedure for the Heck coupling. In a 10 ml round bottomed flask, equipped with a magnetic bar, sulfonate (**1-4**, 0.5 mmol), perfluorohaloarene (**5-7**, 0.5 mmol), K_2CO_3 (or TBAOH, 1 mmol), Pd acetate (or Pd-colloids, 3 mol%) and PPh_3 (6 mol%) in 5 ml of DMF (DMA, NMP or water) were heated at 120 °C for 4 h. At the end of the reaction (GC-MS), the mixture was washed with HCl 5% and extracted with dichloromethane. After drying and evaporation of the solvent *in vacuo*, the products were purified by silica gel column chromatography (eluent petroleum ether/dichloromethane).

Table S1. Example of optimization of reaction conditions in the Heck coupling of vinyl sulphonate **1** with pentafluoroiodobenzene.

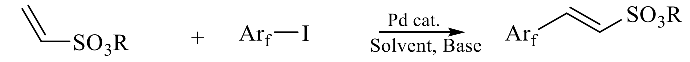

1. R= CH_3 **5**. $Ar_f = C_6F_5$ **8**

Run	Solvent	Base	Pd$_{cat}$	Convn. (%)^b
1	DMF	NaOAc	$Pd(OAc)_2/PPh_3$	-
2	DMF	K_2CO_3	$Pd(OAc)_2/PPh_3$	traces
3	DMF	K_2CO_3	Pd$_{colloids}$^{c}	-
4	DMF	TBAA	Pd$_{colloids}$^{c}	-
5	DMF	K_2CO_3	$Pd(OAc)_2/PPh_3$	-^{d}
6	DMF	K_2CO_3	$Pd(OAc)_2/PPh_3$	traces^e
7	NMP	NaOAc	$Pd(OAc)_2/PPh_3$	-
8	NMP	K_2CO_3	$Pd(OAc)_2/PPh_3$	traces
9	NMP	K_2CO_3	Pd$_{colloids}$^{c}	-
10	NMP	TBAA	Pd$_{colloids}$^{c}	-
11	NMP	K_2CO_3	$Pd(OAc)_2/PPh_3$	-^{d}
12	NMP	K_2CO_3	$Pd(OAc)_2/PPh_3$	-
13^d	H_2O	TBAOH	$Pd(OAc)_2$	by products
14	TBAB	K_2CO_3	$Pd(OAc)_2/PPh_3$	-

15	TBAB	TBAA	$Pd_{colloids}$	by products
16	TBAA	TBAA	$Pd_{colloids}$	by products
17	DMF	Cs_2CO_3	$Pd(OAc)_2/PPh_3$	traces
18	DMA	K_2CO_3	$Pd(OAc)_2/PPh_3$	-
19	DMSO	K_2CO_3	$Pd(OAc)_2/PPh_3$	-
20	Toluene	K_2CO_3	$Pd(OAc)_2/PPh_3$	-

[a] Reaction conditions: sulfonate (0.5 mmol), perfluorohaloarene (0.5 mmol), base (1 mmol), Pd acetate (or Pdcolloids, 3 mol%) and PPh_3 (6 mol%) in 5 ml of proper solvent were heated at 120 °C for 8 h. [b] Yields were evaluated by ^{1}H-NMR of the reaction mixture or GC-MS. [c] Pd colloids prepared *ex situ* from Pd acetate and TBAA. (TBAA= tetrabutylammonium acetate; TBAB= tetrabutylammonium bromide; TBAOH= tetrabutylammonium hydroxide). [d] Reaction performed at 90 °C. [e] Evaluated after 24h.

By means of this procedure, phenyl vinylsulfonate **2** and 4-perfluorooctyl bromo benzene **7** were coupled affording *(E)-phenyl 2-(4-perfluorooctyl)phenylethene sulfonate* **14** in a 65% of yield (pale yellow solid. m.p.: 47-51 °C) and identified by ^{1}H-NMR (400 MHz, $CDCl_3$), δ: 6.96 (d, 1 H, J= 16.8 Hz, alpha vinyl proton); 7.20-7.42 (m, 5 H, aryl protons SO_3Ph), 7.55 (d, 1 H, J= 16.8 Hz, beta vinyl proton); 7.55 (d, 2 H, J= 8.1 Hz, aryl protons); 7.66 (d, 2 H, J= 8.1 Hz, aryl protons). HRMS (ESI-TOF) m/z $[M+H]^+$: calcd for $C_{22}H_{12}F_{17}O_3S^+$ 679.0230, found 679.0238.

Among the expected products, ester **14** was the sole product successfully isolated. In contrast, esters **8**,**9**,**12**,**13** were detected only in trace amounts by GC-MS into the reaction mixture, while sodium salts **10** and **11** were identified by ^{1}H-NMR into the reaction mixture in 30-50% of yield (see fig. S2), but any attempt to isolate them by neutralization/extraction (as sulfonic acids) failed, maybe due to electrophilic addition to the double bond.

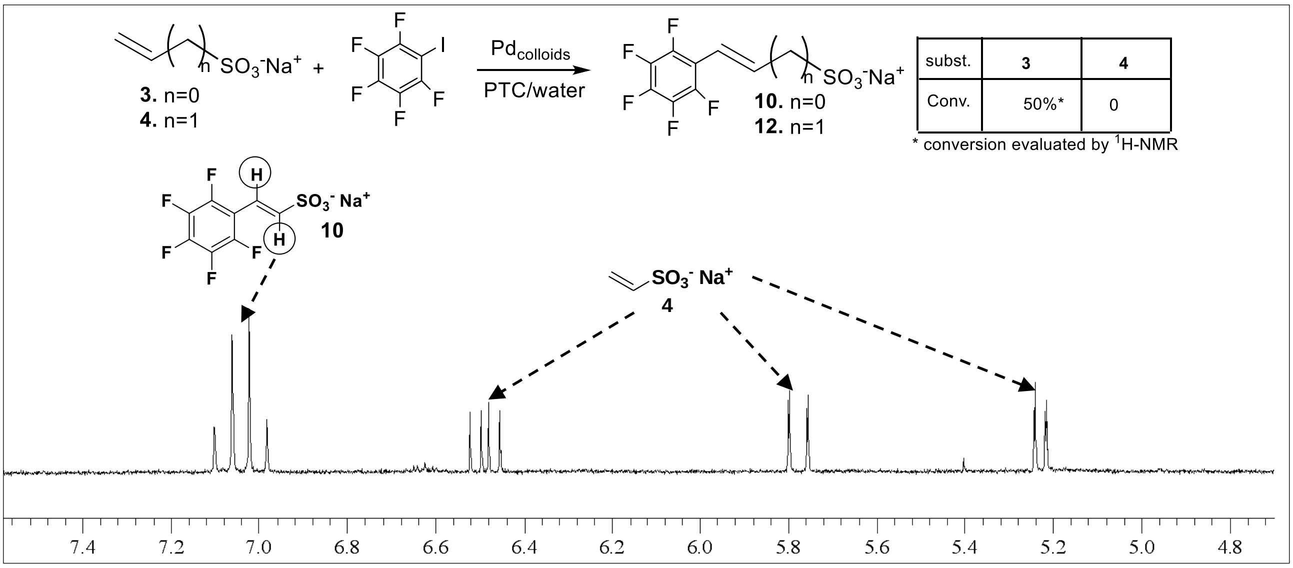

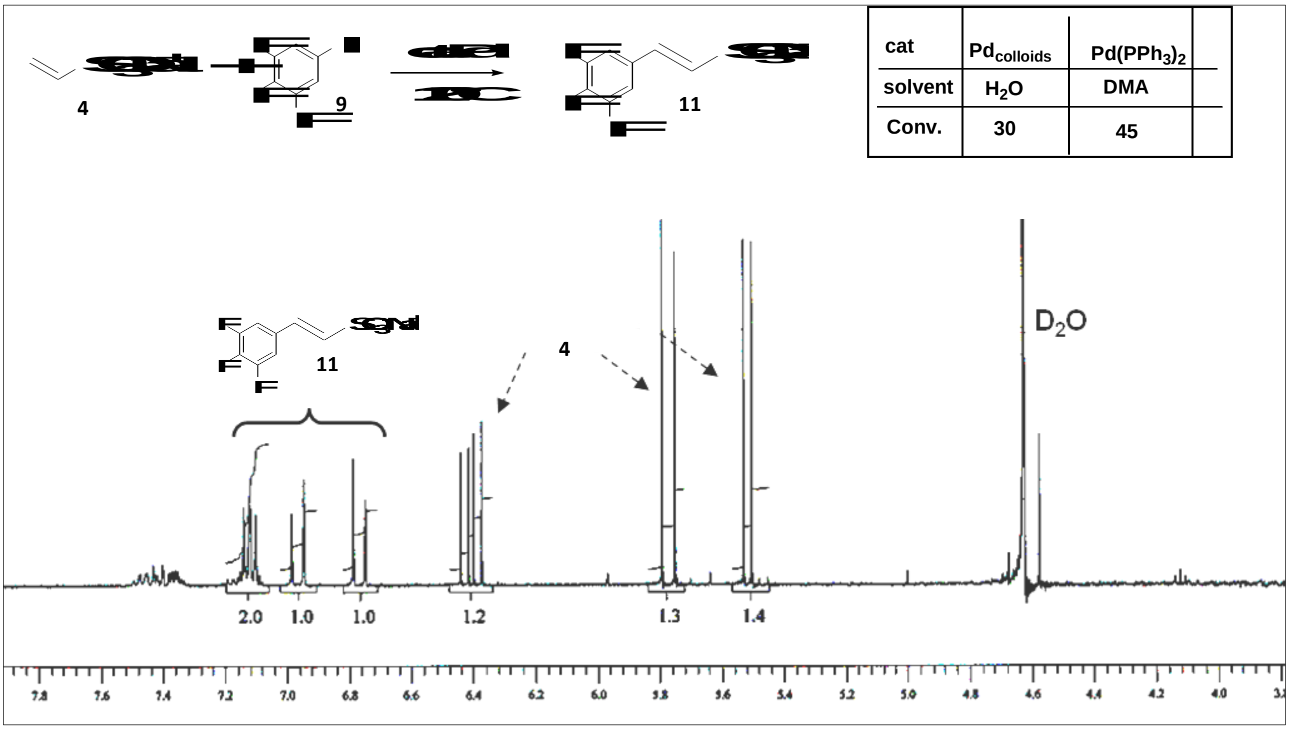

Figure S2. ^{1}H-NMR spectra of reaction mixtures showing the formation of sulfonate salts **10-11**.

S.4 Procedure for Heck-type preparation of carboxylic esters 16 and 19 (eq. 1-2)

In a 100 ml round bottomed flask, equipped with a condenser and a magnetic bar, 1,2,3,4,5-pentafluoro-6-iodobenzene **5** (or methyl 4-iodobenzoate **18**, 5 mmol), methyl acrylate **15** (or 1,2,3,4,5-pentafluoro-6-vinylbenzene **17**, 5 mmol), K_2CO_3 (10 mmol), Pd acetate (3 mol%) and PPh_3 (6 mol%) were refluxed in 50 ml of DMF. At the end of the reaction (GC-MS, 2 ÷ 4 hours), the mixture was washed with HCl 5% and extracted with dichloromethane. After drying and evaporation of the solvent *in vacuo*, the products were purified by silica gel column chromatography (eluent petroleum ether/dichloromethane).

*(E)-methyl 3-(perfluorophenyl)acrylate (**16**)*. Dark red oil, b.p. 95 °C (15 mmHg) (yield 70 %). ^{1}H-NMR (400 MHz, $CDCl_3$), δ: 3.79 (s, 3H, methoxy); 6.70 (d, 1 H, J= 16.5 Hz, alpha vinyl proton); 7.58 (d, 1 H, J= 16.5 Hz, beta vinyl proton); ^{13}C-NMR (400 MHz, $CDCl_3$), δ: 52.2 (MeO), 110.0 (t, $^{2}J_{C,F}$ = 13 Hz), 126.0 (t, $^{3}J_{C,F}$ = 9 Hz), 128.5, 137.95 (d, $^{1}J_{C,F}$ = 253 Hz); 141.81 (d, $^{1}J_{C,F}$ = 258 Hz); 145.75 (d, $^{1}J_{C,F}$ = 254 Hz); 166.52 (carbonyl). ^{19}F-NMR (400 MHz, $CDCl_3$), d: -148.05 (dd, 2F, $^{3}J_{F,F}$ = 21 and $^{4}J_{F,F}$ = 6 Hz), -159.78 (t, 1F, $^{3}J_{F,F}$ = 21 Hz), -170.29 (td like, 2F, $^{3}J_{F,F}$ = 21 and $^{4}J_{F,F}$ = 6 Hz); MS (EI): 252 (M^+, 41), 221 (100), 193 (64), 143 (38), 123 (18), 117 (9), 93 (5), 59 (2). HRMS (ESI-TOF) m/z $[M+H]^+$: calcd for $C_{10}H_6F_5O_2^+$ 253.0282, found 253.0271.

*(E)-methyl 4-(perfluorostyryl)benzoate (**19**)*. This ester was obtained ad white solid (m.p. 133-136 °C) in a 78% of yield. The product was identified by means of comparison of spectral data with the literature. ^{1}H-NMR (400 MHz, $CDCl_3$) lit.[2], δ: 3.90 (s, 3H, methoxy); 7.02 (d, 1 H, J= 16.9 Hz, vinyl proton); 7.40 (d, 1 H, J= 16.9 Hz, vinyl proton); 7.53 (d, 2 H, J= 8.2 Hz, aryl protons); 8.00 (d, 2 H, J= 8.2 Hz, aryl protons); MS (EI): 328 (M^+, 72), 297 (100), 269 (18), 250 (18), 219 (51), 199 (5), 192 (4), 148 (9), 109 (14), 77 (3), 51 (4). HRMS (ESI-TOF) m/z $[M+H]^+$: calcd for $C_{16}H_{10}F_5O_2^+$ 329.0595, found 329.0590.

S.5 Procedure for oxidative Heck coupling: synthesis of phosphonyl ester 22 (scheme 4, eq. 3).

This ester was prepared according to the literature (ref. 31). In a 25 mL round bottomed flask were added $Pd(OAc)_2$ (10 mol%) and Ag_2CO_3 (2.0 equiv) under N_2, followed by DMF (2.4 mL) and DMSO (5%, 120 μL) with stirring. Pentafluorobenzene **21** (0.6 mmol, 1.0 equiv) and dimethyl vinylphosphonate **20** (2.0–3.0 equiv) were added subsequently. The mixture was heated at 120 °C (oil bath). After stirring for 12 h, the reaction mixture was cooled to room temperature and diluted with ethyl acetate, washed with 1 N HCl and brine,

[2] Z. Dogan, R. Paulini, J. A. Rojas Stütz, S. Narayanan, C. Richert *J. Am. Chem. Soc.* **2004**, *126*, 15, 4762–4763

dried over Na_2SO_4, filtered and concentrated. The residue was purified with silica gel chromatography (petroleum ether:ethyl acetate 3:2 as eluent) to provide the pure product *(E)-dimethyl 2-(perfluorophenyl)vinylphosphonate* **22** as colorless oil (b.p. 105 °C, 0.001 mmHg). ^{1}H NMR (400 MHz, $CDCl_3$) δ 3.74 (s, 3H), 3.76 (s, 3H), 6.56 (t, J = 17.4 Hz, 1H), 7.40 (dd, J = 24.2 Hz, 17.4 Hz, 1H). ^{13}C NMR δ 52.84, 52.89, 110.38 (m), 123.01 (dt, J = 188.5 and 8.4 Hz), 132.78 (d, J = 6.1 Hz), 137.95 (dm, $^1J_{C,F}$ = 254.8), 141.95 (dm, $^1J_{C,F}$ = 257.0 Hz), 145.77 (dm, $^1J_{C,F}$ = 255.6 Hz). ^{19}F NMR δ -140.0 (dd, $^3J_{F,F}$ = 22.0 Hz, $^4J_{F,F}$ = 7.3 Hz, 2F), -150.3 (t, $^3J_{F,F}$ = 19.5 Hz, 1F), -160.88 (td, $^3J_{F,F}$ = 19.5 and $^4J_{F,F}$ = 7.3 Hz, 2F). MS (EI) 302 (M^+, 24), 283 (28), 207 (100), 170 (79), 143 (39), 110 (78), 93 (61). HRMS (ESI-TOF) m/z $[M+H]^+$: calcd for $C_{10}H_9F_5O_3P^+$ 303.0204, found 303.0211.

S.6 Procedures for the nucleophilic substitution: synthesis of perfluorinated sulfonic esters 25 and 27

Methyl perfluorohex-2-ene-1-sulfonate **25**. This ester was prepared according to the synthetic scheme depicted below[3]:

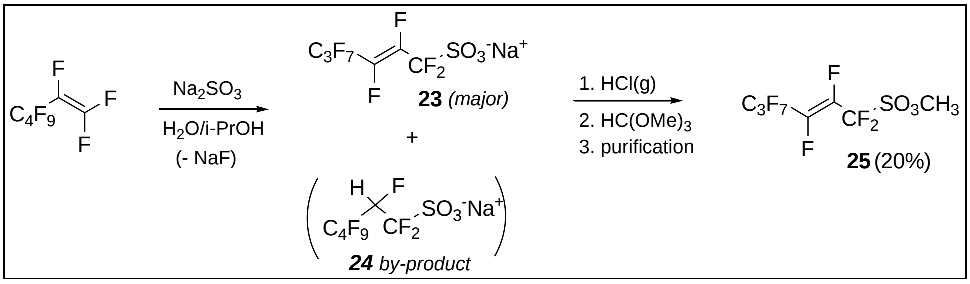

In a typical procedure, a 100 ml round bottomed flask, equipped with a magnetic bar, was charged with 2.17 g (17 mmol) of sodium sulfite and dissolved in 20 ml of distilled H_2O. Then, 900 milligrams (3 mmol) of perfluorohex-1-ene and 70 ml of isopropanol were added. The mixture was refluxed overnight, and after removal of the solvents *in vacuo*, the solid residue was extracted with 40 ml of ethanol in a Soxhlet apparatus for 20 hours. The extracted mixture, which contains the sodium salt **23** (major product) together with its saturated by-product **24** (as revealed by ^{19}F-NMR, see fig. 1), was dissolved in ethanol (50 ml) and treated, under stirring at room temperature, with gaseous HCl for 2 hours until the disappearance of the starting salts (^{19}F NMR). After removal of the by-product (NaCl) by filtration, the solvent was evaporated in vacuo affording to the mixture of the corresponding sulfonic acids. Then, the mixture was dissolved in 50 ml of trimethyl orthoformate and stirred for 48 hours at room temperature to give the mixture of the corresponding esters.

A care distillation of this mixture, first at room pressure to remove the excess of trimethylortoformate, and then with a Kügelrohr apparatus afforded 267 mg (20%) of the most abundant ester *methyl perfluorohex-2-ene-1-sulfonate* **25** as a red oil (b.p. 90 °C, 0.2 mmHg). ^{19}F-NMR (CDCl3) δ ppm (relative to $CFCl_3$) : - 81.20 (t, 3F, $^3J_{F,F}$ = 8.20 Hz, <u>CF_3</u>); - 107.22 (dd, 2F, $^3J_{F,F}$ = 24.4 and $^4J_{F,F}$ = 12.2 Hz, <u>CF_2</u>-SO_3), - 119.60 (m, 2F, CF_2-<u>CF_2</u>-CF=), - 128.50 (m, 2F, <u>CF_2</u>-CF_2-CF=), - 152.50 (dt, 1F, $^3J_{F,F}$ = 143.4 and $^3J_{F,F}$ = 24.4 Hz, =<u>CF</u>-CF_2-SO_3), - 153.70 (m, 1F, <u>CF</u>=CF-CF_2-SO_3). HRMS (ESI-TOF) m/z $[M+H]^+$: calcd for $C_7H_4F_{11}O_3S^+$ 376.9705, found 376.9712.

Methyl 3,4,4-trifluorobut-3-ene-1-sulfonate **27**.

This ester was prepared according to the synthetic scheme depicted below:

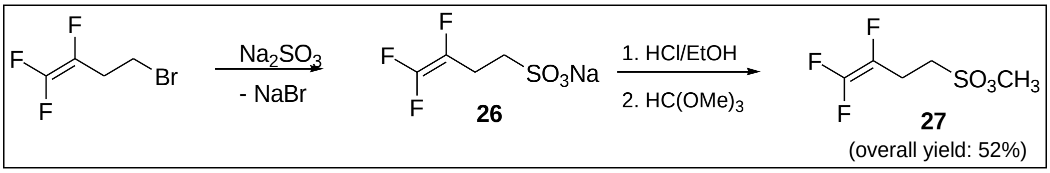

In a 100 ml round bottomed flask, equipped with a condenser and a magnetic bar, 4-bromo-1,1,2-trifluorobut-1-ene (5 g, 26 mmol), Na_2SO_3 (39 mmol), were suspended in a 60 ml of a mixture of water/acetone (5:1), and refluxed overnight. The solvent was then evaporated under vacuum and the residue was dissolved with hot

[3] Gross, U.; Engler, G. *Journal of Fluorine Chemistry* **1985**, *29(4)*, 425-30

isopropanol and filtered to remove the inorganic salts. After that, isopropanol was removed *in vacuo* obtaining sodium 3,4,4-trifluorobut-3-ene-1-sulfonate **26** as white solid (yield 58% after recrystallization from H_2O/ethanol). ^{1}H-NMR (D_2O) δ ppm : 2.50-2.64 (m, 2 H, =CHF-C$\underline{H}_2$), 2.93 (t, 2 H, J= 7.3 Hz, -C$\underline{H}_2$-SO_3CH_3). ^{13}C-NMR (D_2O) δ ppm : 21.42 (d, $^2J_{C,F}$ = 22.9 Hz, =CHF-$\underline{C}H_2$), 46.48 (s, -$\underline{C}H_2$-SO_3Na), 127.41 (ddd, $^1J_{C,F}$ = 231.9 Hz, $^2J_{C,F}$ = 53.4 and 16.8 Hz, F_2C=$\underline{C}$HF-), 153.17 (ddd, $^1J_{C,F}$ = 285.3 and 273.1 Hz, $^2J_{C,F}$ = 47.3 Hz, $F_2\underline{C}$=CHF-). ^{19}F-NMR (D_2O) δ ppm : -190.20 (ddt, 1 F, $^3J_{F,F}$ = 112.9 and 33.6 Hz, $^3J_{F,H}$ = 21.4 =C$\underline{F}$-CH_2), -137.47 (dd, 1 F, $^2J_{F,F}$ = 116.0, $^3J_{F,H}$ = 85.4, C$\underline{F}_2$=CHF-), -118.85 (dd, 1 F, $^2J_{F,F}$ = 85.4, $^3J_{F,F}$ = 33.6, C$\underline{F}_2$=CHF-).
Next, this sodium salt was dissolved in methanol and gaseous HCl was bubbled into the solution for 2 hours. After filtration and evaporation of the methanol, 3,4,4-trifluorobut-3-ene 1-sulfonic acid was obtained as a yellow oil. ^{1}H-NMR (D_2O) 2.65 (t, 2H, *J*= 7.3 Hz, $\underline{CH_2}$-SO_3H), 2.31 (m, 2H, $\underline{CH_2}$-CH_2-SO_3H). ^{19}F-NMR ($CDCl_3$) δ ppm (relative to CF_3COOH) : - 116.80 (dd, 1F, $^2J_{F,F}$ = 88.5 and $^3J_{F,F}$ = 33.5 Hz, C$\underline{F}$F=CFCH_2); -135.50 (dd, 1F, $^2J_{F,F}$ = 88.5 and $^3J_{F,F}$ = 112.9 Hz, CF$\underline{F}$=CFCH_2); - 188.10 (ddt, 1F, $^3J_{F,F}$ = 112.9 Hz, $^3J_{F,F}$ = 33.5 Hz, $^3J_{F,H}$ = 21.4 Hz, CFF=C$\underline{F}$$CH_2$). Then, 50 mL of trimethyl orthoformate were added to the sulfonic acid and the solution was stirred overnight at room temperature. The methylating agent is removed off by distillation under vacuum, leaving the desired *methyl 3,4,4-trifluorobut-3-ene-1-sulfonate 27* as a dark red oil in 89% of yield. ^{1}H-NMR ($CDCl_3$) δ ppm : 2.67-2.85 (m, 2 H, =CHF-C$\underline{H}_2$), 3.24 (t, 2 H, *J*= 7.3 Hz, -C$\underline{H}_2$-SO_3CH_3), 3.83 (s, 3 H, SO_3CH_3). ^{13}C-NMR ($CDCl_3$) δ ppm : 21.05 (d, $^2J_{C,F}$ = 22.9 Hz, =CHF-$\underline{C}H_2$), 41.13 (s, -$\underline{C}H_2$-SO_3CH_3), 55.96 (s, SO_3CH_3). 125.53 (ddd, $^1J_{C,F}$ = 235.0 Hz, $^2J_{C,F}$ = 53.4 and 18.3 Hz, F_2C=$\underline{C}$HF-), 153.21 (ddd, $^1J_{C,F}$ = 288.4 and 276.2 Hz, $^2J_{C,F}$ = 45.0 Hz, $F_2\underline{C}$=CHF-). ^{19}F-NMR ($CDCl_3$) δ ppm : -190.45 (ddt, 1 F, $^3J_{F,F}$ = 116.0 and 33.6 Hz, $^3J_{F,H}$ = 21.4 =C$\underline{F}$-CH_2), - 135.73 (dd, 1 F, $^2J_{F,F}$ = 116.0, $^3J_{F,H}$ = 82.4, C$\underline{F}_2$=CHF-), - 116.65 (dd, 1 F, $^2J_{F,F}$ = 82.4, $^3J_{F,F}$ = 33.6, C$\underline{F}_2$=CHF-). HRMS (ESI-TOF) m/z $[M+H]^+$: calcd for $C_5H_8F_3O_3S^+$ 205.0141, found 205.0149.

S.7 Procedures for Wittig-Horner reaction: synthesis of perfluorinated sulfonic esters 29 and 30

These esters were prepared by adapting a literature procedure[4] that exploits the Wittig-Horner reaction according to the following strategy:

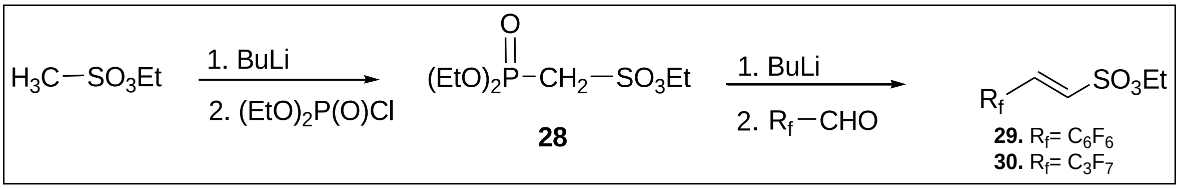

Ethyl diethylphosphoryl methanesulphonate 28. A solution of ethyl methanesulphonate (4.27 mL, 40.3 mmol) in 100 mL of dry THF was treated at -78°C under nitrogen atmosphere with 2.3 M n-BuLi in hexane (19.3 mL, 44.4 mmol). After 15 min., ethyl chlorophosphate (6.6 mL, 44.4 mmol) was added. The solution was kept at -78 °C for 30 min. and allowed to stay at -50°C for 60 min.. Then, 4.4 M NH_4Cl was added (11.0 mL, 44.4 mmol) and the mixture was warmed to room temperature. The mixture was concentrated *in vacuo* to eliminate THF. Then the residue was diluted with 50 mL of water and extracted with dichloromethane (3 x 80 mL). The organic layers were dried over $MgSO_4$ and evaporated to afford ester **28** as crude oil. After distillation, the product was isolated as yellow oil (b.p. 120 °C, 0.1 mmHg) in 60% of yield. ^{1}H NMR ($CDCl_3$) δ : 1.34 (t, J= 6.9 Hz, 6 H); 1.40 (t, J= 7.1 Hz, 3 H), 3.69 (d, J= 17.2 Hz, 2 H), 4.21 (quintet, J= 7.5, 4 H); 4.38 (q, J= 6.9, 2 H). ^{13}C NMR δ 15.24, 16.46, 47.80 (d, *J*= 140.4 Hz), 63.9 (d, *J*= 6.1 Hz), 68.59.
(E)-ethyl 2-(perfluorophenyl)ethenesulfonate 29. A solution of ethyl diethylphosphoryl methanesulphonate **28** (1.0 equiv) in dry THF (1 mL x 0.25 mmol of **28**) was treated at -78°C under nitrogen atmosphere with 2.3 M n-BuLi in hexane (approximately 1.05 equiv.). Stirring was continued for 20 min. Then, freshly pentafuorobenzaldehyde (1.1 equiv.) was added. After an additional 45 min. at -78°C the solution was allowed to warm to room temperature and stirring was continued for 60 hours. The bulk of the solvents was evaporated and the residue was treated with water (5 mL x 0.25 mmol) and extracted with dichloromethane (3 x 5 mL x 0.25 mmol). The organic layers were dried over $MgSO_4$ and evaporated to afford crude unsaturated sulfonyl ester, which was purified by column chromatography (petroleum ether:ethyl acetate 10:1 as eluent) affording pure (E)-ethyl 2-(perfluorophenyl)ethene sulfonate **29** as white solid (m.p. 65 °C) in a 70% of yield. ^{1}H NMR

[4] Carretero, J.C.; Oemillequand, M.; Ghosez, L. *Tetrahedron* **1987**, *43*, 5125-5134.

($CDCl_3$): 1.40 (t, J= 6.9 Hz, 3 H, CH_3); 4.25 (q, J= 6.9, 2 H, CH_2); 7.09 (d, J= 15.9, 1 H, vinyl CH); 7.56 (d, J= 15.9, 1 H, vinyl CH). ^{19}F NMR ($CDCl_3$): -139.7 (dt, $^3J_{F,F}$ = 24.4 and $^4J_{F,F}$ = 6.1 Hz, 2 F), -149.6 (t, $^3J_{F,F}$ = 21.3 Hz, 1 F), -161.8 (m, 2 F). HRMS (ESI-TOF) m/z [M+H]$^+$: calcd for $C_{10}H_8F_5O_3S^+$ 303.0109, found 303.0116.

(E)-ethyl 3,3,4,4,5,5,5-heptafluoropent-1-ene-1-sulfonate ***30***. This perfluorinated sulfonic ester was prepared by an analogous procedure from **28** and 2,2,3,3,4,4,4-heptafluorobutanal (CH_2Cl_2:petroleum ether 7:3 as eluent) in a 60% of yield. The ester was initially obtained as mixture of E/Z isomer 85/15 in ratio. After distillation (b.p. 135 °C, 10 mmHg), the major E isomer was isolated as an oil. ^{1}H NMR ($CDCl_3$): 1.39 (t, J = 7.1 Hz, CH_3), 4.26 (q, J = 7.1, 2 H, CH_2), 6.78 (dt, *J*= 15.4 and 11.2 Hz, vinyl CH), 6.96 (dt, J = 15.4 1.5 Hz, 1 H, vinyl CH). ^{13}C δ 14.95, 69.01, 108.40 (tm, $^1J_{C,F}$ = 264.7 Hz, $CF_3\underline{CF_2}$-), 112.80 (tt, $^1J_{C,F}$ = 255.6 and $^2J_{C,F}$ = 32.0 Hz, $\underline{CF_2}$C=), 117.52 (qt, $^1J_{C,F}$ = 287.6 and $^2J_{C,F}$ = 33.5 Hz, CF_3), 130.4 (t, $^2J_{C,F}$ = 25.2 Hz), 135.8 (t, $^3J_{C,F}$ = 8.4 Hz). ^{19}F NMR ($CDCl_3$): -80.7 (t, $^3J_{F,F}$ = 9.2 Hz, 3 F); -114.9 (quintet, $^3J_{F,F}$ = 9.2 Hz, 2 F); -127.6 (m, 2 F); HRMS (ESI-TOF) m/z [M+H]$^+$: calcd for $C_7H_8F_7O_3S^+$ 305.0077, found 305.0071.
